# Management of type 2 diabetes in the new era

**DOI:** 10.1007/s42000-023-00488-w

**Published:** 2023-09-13

**Authors:** Aris Liakos, Thomas Karagiannis, Ioannis Avgerinos, Konstantinos Malandris, Apostolos Tsapas, Eleni Bekiari

**Affiliations:** 1 Clinical Research and Evidence Based Medicine Unit, Second Medical Department, Aristotle University Thessaloniki, Ippokratio General Hospital, Konstantinoupoleos 49, 54642 Thessaloniki, Greece; 2 Diabetes Center, Second Medical Department, Aristotle University Thessaloniki, 54642 Thessaloniki, Greece; 3 Harris Manchester College, University of Oxford, Oxford, OX1 3TD UK

**Keywords:** Type 2 diabetes, Obesity, Glucagon-like peptide 1 receptor agonists, Sodium-glucose cotransporter 2 inhibitors, Cardiovascular outcome trials

## Abstract

**Purpose:**

Management of type 2 diabetes is advancing beyond glycemic control and is increasingly based on cardiovascular risk stratification. This review summarizes recent advances in the field and identifies existing knowledge gaps and areas of ongoing research.

**Methods:**

A bibliographic search was carried out in PubMed for recently published cardiorenal outcome trials, relevant guidelines, and studies on antidiabetic agents in the pipeline.

**Results:**

Findings from cardiovascular outcome trials support the use of glucagon-like peptide 1 (GLP-1) receptor agonists or sodium-glucose cotransporter 2 (SGLT-2) inhibitors for patients with established cardiovascular disease or multiple risk factors, although it as yet remains uncertain whether the benefits are transferable to patients at lower absolute cardiovascular risk. Additionally, robust evidence suggests that SGLT-2 inhibitors improve clinical outcomes for people with concomitant heart failure or chronic kidney disease. Gut hormone multiagonists will likely represent another major addition to the therapeutic armamentarium for morbidly obese individuals with diabetes. Moreover, nonalcoholic fatty liver disease is a common comorbidity and several liver outcome trials are awaited with great interest. Use of insulin as first-line injectable therapy has been displaced by GLP-1 receptor agonists. Once-weekly formulations of basal insulins along with combinations with GLP-1 receptor agonists are also under development and could increase patient convenience. Technologies of glucose sensors are rapidly evolving and have the potential to reduce the burden of frequent blood glucose measurements, mainly for patients treated with intensified insulin regimens.

**Conclusion:**

Management of type 2 diabetes requires a holistic approach and recent breakthroughs are expected to improve the quality of care.

## Introduction

Maintaining normoglycemia has been the primary focus in the pharmacological management of type 2 diabetes for a very long time. Accordingly, practicing clinicians largely relied on hemoglobin A_1c_ (HbA_1c_) to initiate or modify antidiabetic treatment. In this context, hypoglycemia was the main limiting factor of first-generation antidiabetic agents, such as sulfonylureas and insulin. In the following years, new antihyperglycemic agents were gradually introduced in clinical practice, including the dipeptidyl-peptidase 4 (DPP-4) inhibitors, which have a safe profile in terms of hypoglycemia. Newer insulin regimens that closely mimic physiologic response, such as basal insulin degludec and glargine U-300 as well as the fast-acting insulin aspart (FIAsp) and ultra-rapid lispro (URLi) along with advances in insulin injection devices, also simplified insulin therapy while reducing the risk of hypoglycemia [1, 2]. However, the landscape as regards management of type 2 diabetes was virtually transformed following publication of the results of a series of cardiovascular outcome trials (CVOTs), which were imposed by the drug regulators back in 2008 in response to safety concerns about rosiglitazone [3]. In 2015, EMPA-REG OUTCOME [4] was the first large randomized controlled trial of this kind that provided robust evidence of the cardiovascular benefits of empagliflozin, and soon thereafter, more CVOTs supported the cardioprotective effects of other sodium-glucose cotransporter 2 (SGLT-2) inhibitors and certain glucagon-like peptide 1 (GLP-1) receptor agonists. The salutary effects of the aforementioned drug classes on hard cardiovascular endpoints were more pronounced for patients with established cardiovascular disease (CVD) or multiple risk factors and likely extend beyond glycemic control. Hence, current management of patients with type 2 diabetes is increasingly based on stratification of cardiovascular risk and, in this regard, SGLT-2 inhibitors and GLP-1 receptor agonists are prioritized for patients at high cardiovascular risk [5]. Moreover, high-quality evidence now suggests that SGLT-2 inhibitors reduce rates of hospitalization for heart failure and ameliorate progression of diabetic kidney disease, and this is also taken into account when making treatment choices for patients with these comorbidities. Finally, rates of obesity are constantly increasing among people with diabetes and contemporary recommendations for the management of the disease has put increased emphasis on use of certain GLP-1 receptors agonists for body weight control, such as liraglutide and semaglutide as well as the recently approved dual glucose-dependent insulinotropic polypeptide (GIP)/GLP-1 receptor agonist tirzepatide which is highly effective in reducing body weight [6].

In the foreseeable future, new agents that are currently under clinical development will possibly be added to the therapeutic armamentarium for type 2 diabetes. Regarding glycemic management, insulin icodec is a basal insulin analog under clinical development with prolonged duration of action that allows for once-weekly administration, thereby further reducing the complexity of insulin therapy while increasing its acceptance [7]. Uptake of technological innovations initially designed for type 1 diabetes is also increasing. Soon, traditional measures of blood glucose control such as HbA_1c_ may become outdated and more widespread use of the ambulatory glucose profile derived from continuous glucose monitoring will help to develop a more personalized treatment plan. Finally, nonalcoholic fatty liver disease (NAFLD) represents a significant comorbidity amongst individuals with diabetes for whom effective interventions beyond lifestyle modifications are lacking. Several randomized trials assessing the effect of antidiabetic agents such as SGLT-2 inhibitors, GLP-1, and dual GIP/GLP-1 receptor agonists on liver outcomes are underway. If the encouraging preliminary findings from these liver outcome trials (LOTs) are corroborated, targeting NAFLD will probably be upgraded as a therapeutic priority in upcoming treatment algorithms for type 2 diabetes.

## Methods

This review highlights recent changes in the management of type 2 diabetes to date and outlines existing challenges and future advances that will likely address unmet needs for a chronic condition that represents a substantial burden not only for individuals but also for healthcare systems and society as a whole (Table 1). We conducted a PubMed search up to July 2023 for recently published cardiorenal outcome trials and evidence syntheses thereof as well as pertinent guidelines for the management of diabetes and its associated comorbidities. Moreover, we scanned pharmaceutical companies’ websites to identify candidate molecules under development that might be introduced in clinical practice in the years to come.

**Table 1:** Summary of recent progress in the care of people with type 2 diabetes as of 2023, unfilled knowledge gaps, and areas of ongoing research

Aspect of care	Key developments, existing challenges, and potential future advances
Goal of therapy	• Shift from glucose-centered care to mitigation of cardiovascular complications
Choice of antidiabetic agent	• The primacy of metformin is questioned • Agents with proven cardiorenal benefits (i.e., certain GLP-1 receptor agonists and SGLT-2 inhibitors) should be prioritized for individuals with atherosclerotic CVD, heart failure, or chronic kidney disease as well as indicators of high cardiovascular risk • Uncertainty about choice of appropriate antidiabetic drug for people at low absolute cardiovascular risk
Management of obesity	• GLP-1 receptor agonists and especially semaglutide as well as the dual GIP/GLP-1 receptor agonist tirzepatide are the most effective agents available for body weight reduction • Novel molecules in the pipeline, such as the GIP, GLP-1 and glucagon receptor triple agonist retatrutide, and oral, nonpeptide GLP-1 receptor agonists, such as orforglipron, could induce substantial weight loss that is comparable to bariatric surgery.
Lipid profile	• Constantly decreasing LDL-C targets to < 100 mg/dl for moderate-risk patients with diabetes and as low as 70 and 55 mg/dl for people with multiple atherosclerotic CVD factors or for secondary prevention, respectively
Insulin therapy	• Insulin is regarded as second-line injectable therapy and a GLP-1 receptor agonist should be initiated first • Introduction of more convenient ultra-rapid-acting prandial insulins and basal insulin formulations that will allow for once-weekly administration (e.g., icodec and icosema) might improve adherence, although the net clinical benefits are likely negligible • Disparities remain and access to novel insulin analogs should become more affordable
Monitoring response to therapy	• The ambulatory glucose profile derived from glucose sensors can be used to develop a more personalized treatment plan and might gradually displace traditional indices such as hemoglobin A_1c_

*GLP-1* glucagon-like peptide 1, *SLGT-2* sodium-glucose cotransporter 2, *CVD* cardiovascular disease, *GIP* glucose-dependent insulinotropic polypeptide, *LDL-C* low-density lipoprotein cholesterol

### Mitigation of cardiovascular risk

The American Diabetes Association and the European Association for the Study of Diabetes recommend that GLP-1 receptor agonists or SGLT-2 inhibitors should be used for patients with established CVD as well as patients without established CVD but with high-risk indicators, including age ≥ 55 years plus two or more additional risk factors, such as obesity, hypertension, smoking, dyslipidemia, or albuminuria [5]. The European Society of Cardiology questions the primacy of metformin and advocates upfront treatment with a GLP-1 receptor agonist or a SGLT-2 inhibitor for patients at high or very high cardiovascular risk, such as those with established CVD, end organ damage (i.e., proteinuria, estimated glomerular filtration rate (eGFR) < 30 ml/min/1.73 m^2^, left ventricular hypertrophy or retinopathy), and presence of three or more major risk factors as well as patients with diabetes duration ≥ 10 years plus any additional risk factor [8]. For these individuals, the decision to initiate a GLP-1 receptor agonist or a SGLT-2 inhibitor should be independent of background use of metformin or baseline HbA_1c_ [9]. Of note, GLP-1 receptor agonists appear more effective in preventing stroke and should be prioritized for patients with atherosclerotic CVD, whereas SGLT-2 inhibitors are superior in reducing heart failure hospitalizations [10].

Nevertheless, it still remains unclear whether the favorable cardiovascular effects of the aforementioned drug classes are applicable to people with type 2 diabetes at low absolute cardiovascular risk given that this population was excluded from the respective CVOTs [3]. Randomized controlled trials are needed to address the paucity of evidence about the cardioprotective effects of glucose-lowering medications for this low-risk subgroup, although fewer cardiovascular events are expected and, hence, large sample sizes will be required to effectively capture differences among antidiabetic agents. The remaining knowledge gap concerns a significant number of patients with type 2 diabetes; thus, the conduct of such complex and resource-intensive megatrials is probably less appealing to the ongoing diabetes research enterprise. Registry-based randomized trials, which rely on routinely collected healthcare data for the ascertainment of the outcome, can be proposed to rectify this issue since they allow enrollment of a large sample, which is also representative of a real-world population, at minimal cost. As opposed to observational studies, the randomization protects against the effects of unmeasured confounders and selection bias by indication [11].

To reduce incidence of vascular complications and mortality among patients with type 2 diabetes, a multifactorial approach, apart from glucose regulation, is required taking into consideration the management of hypertension and dyslipidemia [12]. Guidelines for the management of hypertension have not changed substantially in recent years; clinicians should target a systolic blood pressure (SBP) of < 130 mmHg if it can be safely attained, although not < 120 mmHg, as well as a diastolic blood pressure of < 80 mmHg, but not < 70 mmHg, and these blood pressure targets should be individualized. For older people aged > 65 years, a more moderate SBP goal of < 140 mmHg might be appropriate, whereas for patients at increased risk of a cerebrovascular event, such as those with a history of stroke, a SBP of < 130 could be considered. Renin angiotensin aldosterone system (RAAS) blockers are considered first-line antihypertensive therapy for patients with type 2 diabetes, especially in the presence of albuminuria or coronary artery disease, while treatment can be advanced with the addition of a calcium channel blocker or a thiazide like diuretic [8, 12].

Regarding the management of dyslipidemia, low-density lipoprotein cholesterol (LDL-C) targets are constantly on the decrease. Based on the underlying cardiovascular risk, a LDL-C target of < 100 mg/dl is recommended for moderate-risk patients, whereas for patients with multiple atherosclerotic CVD factors or for secondary prevention, LDL-C levels below 70 and 55 mg/dl, respectively, should be aimed for, along with a reduction of at least 50% in LDL-C. In this regard, the majority of patients with type 2 diabetes will eventually qualify for high-intensity statin therapy, such as atorvastatin 40–80 mg or rosuvastatin 20–40 mg: if the target LDL-C level is not achieved stepwise, addition of ezetimibe followed by a proprotein convertase subtilisin/kexin type 9 inhibitor should be considered [13]. Nevertheless, real-world data consistently suggest that control of LDL-C remains decidedly suboptimal in high-risk individuals [14]. Because practicing clinicians are often reluctant to pursue very low LDL-C targets, more efforts are needed to bridge this gap between guideline recommendations and clinical care.

### Management of concomitant heart failure

Heart failure is predicted to be the new epidemic of the twenty-first century. People with type 2 diabetes are at increased risk for developing heart failure, which further increases their risk of adverse outcomes, mainly severe exacerbations that require hospitalization, as well as of mortality. Several trials have documented the effectiveness of SGLT-2 inhibitors for reducing rates of worsening heart failure in individuals across the full range of ejection fraction [15–17]. The beneficial effects of SGLT-2 inhibitors on heart failure outcomes are mediated by osmotic diuresis and occur irrespective of the presence of diabetes, thereby expanding the indication of these agents for patients with isolated heart failure without diabetes. Guidelines for the management of heart failure from the American College of Cardiology/American Heart Association have been modified accordingly and now include strong recommendations in favor of SGLT-2 inhibitors for patients with reduced ejection fraction as well as for individuals with preserved ejection fraction, for whom effective medical therapies are, admittedly, more limited [18].

Recent findings derived from network meta-analysis suggest that GLP-1 receptor agonists probably also reduce hospital admissions for worsening heart failure [19], although, in contrast to SGLT-2 inhibitors, dedicated trials for heart failure outcomes with these agents are lacking. GLP-1 receptor agonists are increasingly being used as a component of obesity treatment, which is clearly a pressing need for patients with comorbid heart failure. However, at the same time these agonists increase heart rate and, hence, any modest clinical benefits might diminish, especially among individuals with severe left ventricular dysfunction. Patients with preserved ejection fraction who have less well-established treatment options could benefit more from weight reduction. In this regard, it might be prudent for future research on GLP-1 receptor agonists to focus primarily on this subpopulation. Indeed, results from the SUMMIT trial (NCT04847557), a study of the newly approved GIP/GLP-1 receptor agonist tirzepatide in people with heart failure with preserved ejection fraction and obesity, could offer new insights.

### Prevention of diabetic kidney disease

Diabetic nephropathy affects as many as 40% of patients with type 2 diabetes and is the leading cause of end-stage kidney disease requiring renal replacement therapy. Interventions to stabilize renal function in patients with diabetic kidney disease include optimal glycemic control, more stringent blood pressure targets, and use of RAAS inhibitors as well as management of excess cardiovascular risk with an appropriate lipid-lowering regimen. Beyond RAAS blockade, based on findings from cardiorenal outcome trials patients with type 2 diabetes and an eGFR < 60 ml/min/1.73 m^2^ or albuminuria defined as an albumin to creatinine ratio ≥ 30 mg/g should preferably be treated with a SGLT-2 inhibitor to reduce the risk of kidney failure [20–22]. Because their effect on blood glucose is modest with worsening renal function owing to the decrease in the filtered glucose load, favorable kidney outcomes with SGLT-2 inhibitors are likely related to a reduction in intraglomerular pressure and are independent of the presence of type 2 diabetes.

GLP-1 receptor agonists are considered second-line therapy for cardiovascular risk reduction in patients with type 2 diabetes and chronic kidney disease (CKD) who do not meet glycemic targets with a SGLT-2 inhibitor or for whom a SGLT-2 inhibitor is contraindicated [5, 23]. Nevertheless, this recommendation is mainly driven by the positive effect of these agents on reducing the risk for persistent macroalbuminuria and evidence for hard renal endpoints is still lacking. In this regard, the FLOW trial (NCT03819153) is a dedicated kidney outcomes trial with semaglutide that is expected to clarify whether this once-weekly GLP-1 receptor agonist delays the progression of kidney disease [24].

Apart from glucose-lowering medications, the nonsteroidal mineralocorticoid receptor antagonist finerenone has recently received regulatory approval for people with type 2 diabetes and concomitant nephropathy with albuminuria. The FIDELIO-DKD trial showed that finerenone ameliorates progression of CKD and reduces rates of cardiovascular events [25]. All participants in this trial received background therapy with RAAS blockers, but only a small minority were treated with a SGLT-2 inhibitor. Hence, the added value of finerenone for kidney protection on top of standard of care therapy with SGLT-2 inhibitors warrants further investigation. Finally, initial promising evidence of renoprotection with endothelin receptor antagonists such as atrasentan should prompt further research to investigate the potential role of this drug class for the treatment of patients with type 2 diabetes at high renal risk [26].

### Development of new GLP-1 receptor agonists

Tirzepatide is the first-in-class dual GIP/GLP-1 receptor agonist with marketing authorization for the treatment of diabetes in Europe and the USA administered once weekly by subcutaneous injection. Compared to GLP-1 receptor monoagonism, combined activation of GLP-1 and GIP appears to have a synergistic effect. In tirzepatide’s clinical development program, SURPASS, the drug was highly effective in reducing HbA_1c_ up to approximately 2% for the maximal approved dose of 15 mg and even outperformed other potent GLP-1 receptor agonists without increasing the risk of hypoglycemia. Moreover, tirzepatide 15 mg was associated with weight loss of up to approximately 9 kg relative to placebo and was also superior in head-to-head comparisons with dulaglutide and semaglutide. The incidence of gastrointestinal side effects was similar to that of GLP-1 receptor agonists [6]. Given the well-documented cardiovascular benefits of certain GLP-1 receptor agonists, evidence of the effect of tirzepatide on long-term, hard clinical endpoints is much anticipated. Initial meta-analytic findings from the SURPASS clinical development program are encouraging [27]. The ongoing SURPASS-CVOT trial (NCT04255433) with more than 13,000 participants, to be completed by the end of 2024, is expected to clarify the cardiovascular effects of tirzepatide compared to dulaglutide.

The continuous refinement of GLP-1 receptor agonists has led to the development of multiagonist peptides that have the potential to reshape the management of obesity and hyperglycemia. In a phase 2 trial involving adults with obesity, the GIP, GLP-1, and glucagon receptor triple agonist retatrutide induced substantial body weight reduction of 24.2% after 48 weeks of intervention [28]. Hopefully, triple peptide hormone receptor agonists could more closely mimic the effects of metabolic surgery, which, though not scalable, offers substantial weight loss benefits and could even lead to remission of diabetes.

An oral formulation of the GLP-1 receptor agonist semaglutide taken once daily has also received marketing authorization. Although less effective for weight reduction, it could offer a more attractive option for earlier initiation of GLP-1 receptor agonist therapy in patients reluctant to use injectable agents [29]. Propitiously, results from the dedicated CVOT for oral semaglutide PIONEER 6 trial suggest a positive impact on all-cause and cardiovascular mortality [30]. Finally. orforglipron is another nonpeptide GLP-1 receptor agonist for daily oral administration for which weight loss up to 14.7% among patients with obesity has been observed in a phase 2 clinical trial [31].

### The “diabesity” epidemic and NAFLD

Liver steatosis, which can progress to nonalcoholic steatohepatitis (NASH) and cirrhosis, is the most common hepatic disorder in Western countries that affects as many as 70% of people with type 2 diabetes, especially those who are overweight or obese. NAFLD represents a major public health problem of growing prevalence for which licensed treatments are lacking. Several antidiabetic agents have been evaluated as candidate molecules for the management of NAFLD [32]. Pioglitazone is associated with reductions in hepatic steatosis and lobular inflammation, while the GLP-1 receptor agonists liraglutide and semaglutide, which have also received marketing authorization at higher doses for chronic weight management, might promote histologic resolution of NASH and halt the progression of fibrosis. Finally, studies using mainly magnetic resonance imaging (MRI)-based techniques for evaluation of liver fat content and fibrosis have also pointed to potential benefits with the use of several SGLT-2 inhibitors. Interestingly, reduction in liver fat content has been noted in a MRI substudy with the dual GIP/GLP-1 receptor agonist tirzepatide [33]; however, SYNERGY-NASH, a dedicated trial with liver histological endpoints (NCT04166773), will provide more specific data on hepatoprotection for this agent.

### Progress in insulin therapy

Many patients with type 2 diabetes will at some point during the course of the disease need insulin. Although insulin therapy has made considerable progress over the last few years, insulin is no longer regarded as first-line injectable therapy for people with type 2 diabetes. Before initiation of insulin, use of GLP-1 receptor agonists should be considered unless contraindicated because of their comparable glycemic efficacy and their favorable profile with respect to hypoglycemia [34]. Moreover, for patients already receiving basal insulin, a GLP-1 receptor agonist should be preferred over prandial insulin. Fixed ratio combinations of basal insulin with GLP-1 receptor agonists are also commercially available that minimize the injection burden while balancing out the risk of hypoglycemia and weight gain [35]. Nevertheless, it is still imperative not to postpone insulin therapy if it is deemed appropriate for certain individuals. Clinician and patient inertia regarding initiation of insulin has long been recognized and the extent to which this phenomenon will be affected by modern perceptions as to the role of insulin in type 2 diabetes pharmacotherapy remains to be elucidated.

The added value of newer basal insulin analogs such as degludec and glargine U-300 for glycemic control has so far been negligible, their main advantages being related to the lower risk of nocturnal hypoglycemia [2]. Regarding prandial insulin, ultra-rapid acting insulin analogs including FIAsp and URLi have recently been introduced in everyday clinical practice. Theoretically, their faster onset of action could better control mealtime glucose excursions and allows for greater dosing flexibility. However, these pharmacokinetic properties have not been shown to translate into clinically relevant benefits regarding the effect on HbA_1c_ or incidence of hypoglycemia compared to their rapid-acting counterparts [1]. All these advancements should be put in context with the steeply rising cost of insulin in the USA and elsewhere which deters compliance and hampers optimal glycemic control. Biosimilars did not have a sizeable impact on cost savings; thus, professional diabetes associations and patient advocacy groups are calling for further reductions in insulin prices and especially out-of-pocket expenses.

Icodec and basal insulin Fc (efsitora alfa) represent novel basal insulin formulations with a pharmacokinetic profile suitable for once-weekly administration. Insulin icodec is currently being evaluated in a series of clinical trials (ONWARDS). Specifically, in a 26-week, phase 2 trial, icodec showed comparable glycemic efficacy to insulin glargine and similar rates of hypoglycemia among patients treated with metformin with or without a DPP-4 inhibitor [7]. In another 26-week, phase 3 study enrolling patients treated with basal insulin, switching to icodec was superior to insulin degludec for reducing HbA_1c_, though with modest weight gain and numerically more episodes of hypoglycemia [36]. Preliminary results also suggest that basal insulin Fc is non-inferior to degludec in terms of HbA_1c_ lowering [37]. Although more research is needed on the optimal titration scheme, the potential introduction in clinical practice of a once-weekly basal insulin regimen could encourage insulin acceptance and improve adherence. Finally, a combination of insulin icodec with the GLP-1 receptor agonist semaglutide (icosema) intended for once-weekly administration is in the pipeline.

### Continuous glucose monitoring and insulin pumps

Self-monitoring of blood glucose in type 2 diabetes has consistently failed to provide clinically meaningful benefits. Use of continuous glucose monitoring (CGM) is widespread among patients with type 1 diabetes but its place in the management of type 2 diabetes and predominantly people treated with intensified insulin regimens remains controversial. HbA_1c_ reductions in the order of 0.3–0.4% have been observed with CGM compared to fingerprick measurements in randomized trials enrolling patients with type 2 diabetes receiving basal insulin alone or multiple daily injections [38, 39], but evidence of the ability to decrease risk of severe hypoglycemia is lacking. In contrast to use of HbA_1c_ to evaluate glycemic control, CGM additionally captures glycemic variability and hypoglycemic episodes and, in this sense, time spent in target range as well as time spent in hypoglycemic range are gradually replacing traditional measures of glycemic efficacy initially in the context of clinical research and potentially in clinical practice as well. Similarly, the effectiveness of continuous subcutaneous insulin infusion has not been convincingly demonstrated in type 2 diabetes. In a large, randomized trial (OpT2mise), patients with type 2 diabetes and inadequate glycemic control achieved a greater reduction by 0.7% in HbA_1c_ with insulin pump therapy compared to multiple daily injections. The daily insulin dose was also lower, but the two groups did not differ in rates of hypoglycemia [40]. Interestingly, extensive research is currently being conducted on sweat-based and other noninvasive, wearable glucose sensors. Although all the aforementioned technologies are attractive tools for the management of insulin-treated patients with type 2 diabetes, their high cost and concerns about the associated user information overload are still barriers to their wider adoption. The promise that such exciting technologies will lead to improvements in patient-oriented outcomes has not yet been realized.

## Conclusions

Recent innovations, including the introduction of antidiabetic drugs with proven cardiorenal benefits, highly effective agents for inducing weight loss, and more convenient insulin regimens and glucose sensors are having a profound impact on the everyday lives of patients with type 2 diabetes. Beyond these exciting interventions, lifestyle modification and diabetes self-management education and support, which are the mainstay of a holistic diabetes care plan, should continue to be energetically promoted.
